# Supplementary material for: Disruption of the psychiatric risk gene Ankyrin 3 enhances microtubule dynamics through GSK3/CRMP2 signaling
Source: Transl Psychiatry. 2018 Jul 25;8:135. doi: 10.1038/s41398-018-0182-y (PMC6060177; doi:10.1038/s41398-018-0182-y)
Supplement: Supplementary file 1 — Supplemental legends [file 41398_2018_182_MOESM1_ESM.docx]

**Supplementary Figure 1.** Expression of *Ank3* in hippocampal tissue from *Ank3*+/+ and *Ank3*+/- mice. qPCR quantification using primers specific for alternative starting exon 1b, exon 1e, exon 1f, and exon 1s revealed approximately 50% reduced expression of brain-specific exon 1b in *Ank3*+/- mice compared to *Ank3*+/+ mice, whereas expression of other *Ank3* exons did not significantly differ. Samples were measured in triplicate and expression was normalized to beta-2-microglobulin. Data are presented as percent of the control group for each exon. Data were compared using two-tailed Student’s *t*-test for each exon. Data are presented as mean ± s.e.m. * *P*<0.05.

**Supplementary Figure 2.** Fourteen sgRNA targeting *Ank3* exon 1b were screened by qPCR for efficiency in reducing exon 1b expression in mouse neuro-2a cells. qPCR quantification was performed using primers specific for alternative starting exon 1b. Three biological replicate samples were measured in triplicate and expression was normalized to beta-2-microglobulin and the control sgRNA (C, control sgRNA). sgRNA #7 was selected as the *Ank3*-targeting sgRNA for subsequent experiments. Data were analyzed using one-way ANOVA followed by Bonferroni *post hoc* tests. Statistical significance is reported for each sgRNA group compared to the control sgRNA. Data are presented as mean ± s.e.m. * *P*<0.05, ** *P*<0.01, ****P*<0.001.

**Supplementary Figure 3.** Brain-specific *Ank3* expression was not changed in mouse neuro-2a cells after treatment with lithium (1mM) or CHIR99021 (1µM) for 1h. qPCR quantification was performed using primers specific for alternative starting exon 1b. Expression was normalized to beta-2-microglobulin and the vehicle treated control sgRNA group. Control, control sgRNA; Ank3, *Ank3*-targeting sgRNA. Three biological replicates for each sample were measured in triplicate. Data are presented as mean ± s.e.m averaged across two independent experiments. n.s. indicates not significant.
