# Supplementary material for: Disruption of the psychiatric risk gene Ankyrin 3 enhances microtubule dynamics through GSK3/CRMP2 signaling
Source: Transl Psychiatry. 2018 Jul 25;8:135. doi: 10.1038/s41398-018-0182-y (PMC6060177; doi:10.1038/s41398-018-0182-y)
Supplement: Supplementary file 2 — Supplementary Information [file 41398_2018_182_MOESM2_ESM.docx]

**Supplementary Information**

**Supplementary Methods**

**Animals**

Male *Ank3*+/- mice with a targeted heterozygous deletion of *Ank3* exon 1b^1^ were crossed to female C57BL/6J mice (Jackson Laboratory, Bar Harbor, ME) to generate *Ank3*+/- and *Ank3*+/+ progeny. Mice were housed 2-5 per cage with same-sex littermates under 12h:12h light-dark cycle (lights on at 7 a.m.), and *ad libitum* access to food and water.

**RNA sequencing and Analysis**

Male mice were decapitated, and the entire head was cooled rapidly in liquid nitrogen. The brain was removed and the hippocampi were dissected, snap frozen in dry ice, and stored at -80°C. RNA was extracted using the AllPrep DNA/RNA mini kit (Qiagen, Valencia, CA, USA). RNA quality and concentration was assessed using the RNA 600 Nano Kit (Agilent Technologies, Santa Clara, CA), which determined that all samples had an RNA integrity number > 8. RNA (50 ng) from each of 10 mice per genotype was pooled for RNA sequencing (Hudson Alpha Institute for Biotechnology, Huntsville, AL) at a depth of 25 million paired reads of 50 bases. Raw sequence reads were trimmed by 15 bases at the 5’ end using the Trimmomatic (v1) tool^2^ to decrease the number of mismatches to the genome in the alignment step of the analysis. Trimmed sequence reads (minimum length 50 bp) were analyzed using the Tuxedo package^3^ within the GenePattern platform (http://genepattern.broadinstitute.org) hosted by the Broad Institute of MIT and Harvard. Using the TopHat module, the reads were first aligned to each other by mapping to the *Mus musculus* GRCm38/mm10 mouse genome, allowing for 2 mismatches per read, a maximum intron length of 500 kb, and maximum insertion/deletion length of 3 bases. The aligned reads were then assembled onto the mouse genome using the Cufflinks module. The Cuffdiff module was then used to identify differentially expressed genes between reads generated from the *Ank3* +/+ and *Ank3* +/- pools. Since hippocampal dissections could include adjacent choroid plexus, as a precaution, we excluded genes that are highly expressed in choroid plexus^4^ from the analyses. Differentially expressed genes that had less at least 2× read coverage, a fold change greater than ± 1.2, and an uncorrected P < 1x10^-3^ were retained for further analyses.

**Primary neuronal culture and transfection**

Postnatal day 0 *Ank3*+/+ and *Ank3*+/- pups were decapitated and the forebrain tissue was dissected, collected, and washed with chilled HBSS (ThermoFisher, Waltham, Ma, Gibco). The collected tissues were incubated with 5% Trypsin-EDTA (ThermoFisher, Waltham, Ma, Gibco) and 0.1 mg/ml DNase I (Sigma-Aldrich, St. Louis, Mo) in HBSS at 37 °C for 15 min. The digested tissues were washed 3 times with HBSS, and were triturated a maximum of 10 times with standard 1mL pipettes. 100-200,000 dissociated cells were plated onto a 35mm glass-bottom cell culture dish (World Precision Instruments, Sarasota, Fl) pre-coated with laminin (2.5 µg/ml, Sigma-Aldrich, St. Louis, Mo) and Poly-L-Lysine (Sigma-Aldrich, St. Louis, Mo). The neurons were cultured and maintained in the Neurobasal A medium (ThermoFisher, Waltham, Ma, Gibco) supplemented with 2% B-27 supplement (ThermoFisher, Waltham, Ma), 0.25% glutamine (ThermoFisher, Waltham, Ma), and 1% Penicillin/Streptomycin (ThermoFisher, Waltham, Ma) at 37 °C and 5% CO2. The mPA-GFP-EB3-7 plasmid (Addgene #57130) was transiently transfected into the primary neurons at DIV 11-12. For the transfection, the culture medium was removed and set aside to be used again after transfection. Each dish of cultured neurons was washed twice with 500 μl pre-warmed Neurobasal A medium (without B27, glutamine and antibiotics). A total of 0.5 μg of mPA-GFP-EB3-7 plasmid DNA and 2.0 μl Lipofectamine 2000 (Invitrogen) were added into 100 μl Neurobasal A (without B27, glutamine and antibiotics) medium and incubated at room temperature for 20 min. The DNA Lipofectamine 2000 mixture was then added dropwise to the culture dish and incubated for 1 h in a 37°C cell culture incubator. Neurons were washed three times with Neurobasal A medium before they were placed back in the conditioned medium (the medium that had been used to culture the neurons before transfection). Transfected neurons were imaged 2-3 d later.

**Live-cell imaging and analysis**

Culture dishes were mounted in a live-cell imaging chamber, maintained at 37 °C and 5% CO2, and imaged in the culture medium. Neurons were imaged with a 60 x /NA1.45 oil objective on a Nikon A1R confocal microscope controlled by the NIS-Elements Advanced Research software with excitation set at 488nm. Axons were identified and distinguished from dendrites by their characteristic morphology (longer length, thin and uniform diameter, sparse branching, and prominent growth cones).^5^ Axon segments 70-150 μm in length were imaged starting ~60 μm from the soma at 2 s intervals for 300 s.^6,7^ Kymographs were generated from the time-lapse images and were analyzed for EB3-GFP comet motility with the Kymolyzer macro for ImageJ.^8^ EB3 comet motility was monitored by manually tracing individual comet trajectories in the kymographs generated from the time-lapse images. The length of the trajectory, the longevity, and the average velocity of the EB3 comets were analyzed.

**Cell culture**

Mouse neuro-2a cells (ATCC, Manassas, VA) were maintained on 10cm plastic cell culture treated dishes in Eagle Minimum Essential Medium (EMEM) supplemented with 10% fetal bovine serum (FBS) at 37°C in a CO_2_ water-jacketed incubator. For individual experiments, the cells were passed into 6-well plates and grown to 75% confluency before transfection. For drug experiments, cells were treated for 1h with lithium (Sigma-Aldrich, St. Louis, Mo) or CHIR99021 (LC Laboratories, Woburn, Ma), or 24h with lacosamide (Sigma-Aldrich, St. Louis, Mo).

**Western blotting**

Mouse tissue: Mice were decapitated and the hippocampus was dissected on ice and homogenized in 0.9% saline containing phosphatase and protease inhibitors (Roche, Indianapolis, In). Lysate was mixed with 2× Laemmli Sample Buffer (BIO-RAD, Hercules, California) and heated to 95°C for 10 min. For analysis, 20μg of total protein was separated on a 12.5% SDS-PAGE gel followed by transfer to a polyvinylidene difluoride (PVDF) membrane. The blots were probed with antibodies against EB3 (ABCAM, Cambridge, Ma, 1:2000) and GAPDH (Cell Signaling, Danvers, MA, 1:2000). Quantification of the Western blots was performed by measuring the band intensity using ImageJ analysis software.^9^

Cultured cells: Cells grown in 6-well tissue culture plates were rinsed briefly with PBS, incubated in RIPA buffer containing 1X Halt^TM^ Protease and Phosphatase Inhibitor Cocktail (ThermoFisher, Waltham, Ma) on ice for 10 min, and homogenized using a sonicator for 20 min at 4°C. After sonication, total protein was quantified using the bicinchoninic acid assay (BCA, ThermoFisher, Waltham, Ma). The samples were mixed in 6× Laemmli’s reducing SDS sample buffer (Boston BioProducts, Ashland, Ma) and RIPA buffer to a final concentration of 0.6μg/μl. For Western blot analysis, 20μg of total protein was separated by SDS-PAGE and processed as described for mouse tissue.

See Supplementary Table 1 for the antibodies used in this study,

**CRISPR/dCas9-mediated transcriptional repression of brain-specific *Ank3***

To accomplish transcriptional repression of brain-specific isoforms of *Ank3*, single guide RNA (sgRNA) sequences were designed to target the genomic sequence within exon 1b using the guide RNA design tool from MIT (http://crispr.mit.edu). Fourteen sgRNA and one non-targeting control sgRNA (Supplementary Table 2) were designed with an NGG protospacer motif and cloned into the lenti_sgRNA(MS2)_EF1α backbone plasmid^10^ using a Golden Gate reaction with BsmBI restriction enzyme and transformed in to Stbl3 chemically competent *e. col I (*ThermoFisher, Waltham, Ma)*.* Each sgRNA plasmid was co-transfected with the pHAGE-EF1α-dCas9-KRAB plasmid (Addgene #50919)^11^ into mouse neuro-2a cells (American Type Culture Collection, Manassas, VA) using a standard protocol and Lipofectamine 2000 (ThermoFisher, Waltham, Ma). Briefly, 4μg of total plasmid DNA was diluted in OPTI-MEM (ThermoFisher, Waltham, Ma, Gibco) with 4μl of Lipofectamine 2000 reagent and incubated at room temperature for 10 min. The solution was then slowly added to the cell culture plate and incubated overnight. The media was changed the next day and the cells grown for 48h, then subjected to positive selection using zeocin (300ug/ml) and puromycin (2μg/ml) for 72h and used for experiments.

**Tubulin Polymerization Assay**

*Ank3* repressed and control mouse Neuro-2a cells were processed to obtain soluble and insoluble protein fractions as described.^12,13^ Briefly, the cells were rinsed once with PBS and soluble tubulin was extracted by adding hypotonic soluble protein extraction buffer (137mM NaCl, 20mM Tris-HCl, 1% Triton X-100, and 10% glycerol, and 1X Halt Protease and Phosphatase Inhibitor Cocktail) to the cells for 2 min while gently rocking the cell culture plates. The buffer was collected and saved on ice as the soluble protein fraction. The cells were briefly rinsed with cold PBS and the insoluble protein fraction was immediately extracted using the polymerized fraction buffer (137mM NaCl, 20mM Tris-HCl, 1% Triton X-100, 10% glycerol, 1% SDS, and 1X Halt Protease and Phosphatase Inhibitor Cocktail). Protein was quantified using the BCA kit (ThermoFisher, Waltham, Ma). The samples were mixed in 6× Laemmli’s reducing SDS sample buffer protein extraction buffer and processed for Western blot to detect alpha-tubulin. Quantification of the Western blots was performed by measuring the band intensity using ImageJ analysis software.^9^

**qPCR**

Total RNA was extracted from mouse hippocampus or neuro-2a cells using the RNeasy Kit (Qiagen, #74106, Venlo, The Netherlands) according to the manufacturer’s instructions. Purified RNA was quantified using a NanoDrop 1000 (ThermoFisher, Waltham, Ma). A total of 1 μg of RNA was used to generate cDNA using qScript cDNA SuperMix (Quanta bio, Beverly, Ma). The first-strand synthesis reaction consisted of 1μg RNA (variable volume), 4μl 5X qScript cDNA SuperMix, and RNase/DNase-free water to a final volume of 20μl. The reaction was placed in a thermal cycler and processed with the following conditions: 25°C for 5 min, 42°C for 30 min, 85°C for 5 min, and then cooled to 4°C. qPCR was performed using SYBR Green Master Mix (Roche Life Science) on a LightCycler 480 thermocycler (Roche Life Science) with gene specific primers (Supplementary Table 3). Each reaction consisted of 500ng cDNA, 5μl 2X LightCycler 480 SYBR Green I Master mix (Roche Diagnostics Corporation, Indianapolis, In) and gene specific primers at a final concentration of 0.5μM each in a 10μl reaction. Once the reaction was mixed, the plates were placed in the LightCycler 480 and processed with the following protocol: preincubation at 95°C for 5 min, followed by 45 cycles of 95°C, 60°C and 72°C for 10 s each, with a single acquisition step after the extension step of each cycle. The relative expression of the samples was obtained by normalizing the Cp values to the beta-2-microglobulin endogenous housekeeping gene.

**Supplementary Results**

**CRISPR/dCas9-mediated transcriptional repression of *Ank3* exon 1b**

We established an *Ank3* repression neuronal model system using CRISPR/dCas9 technology. Multiple sgRNA sequences were designed to target different genomic sites for *Ank3* exon 1b (Supplementary Table 2). A non-targeting sgRNA was used as a control. Mouse neuro-2a cells were dual transfected with the pHAGE-EF1α-dCas9-KRAB plasmid and the sgRNA(MS2)_EF1α_sgRNA plasmid expressing each sgRNA. qPCR validation using primers specific for *Ank3* exon 1b determined that all but one of the 14 sgRNAs achieved statistically significant repression compared to the control sgRNA (Supplementary Figure 2). To validate whether the repression was specific for *Ank3* isoforms containing exon 1b, qPCR was used to measure the expression of each starting exon (exon 1b, exon 1e, exon 1f, and exon 1s) for a subset of selected sgRNAs. While most sgRNAs effectively repressed exon 1b, there was some detectable repression of other starting exons. sgRNA #7 was found to significantly repress exon 1b (F_(4,25)_=4.508; p<0.05) without significant changes to exon 1e, exon 1f, or exon 1s. *Ank3* exon 1b repression by sgRNA #7 was comparable to that of the *Ank3* +/- mice (53% reduction in *Ank3*+/- vs *Ank3*+/+ mice [Supplementary Figure 1] and 60% repression for *Ank3* exon 1b-targeting transcriptional repressor vs control [Supplementary Figure 2]). Therefore, we used sgRNA #7 to repress brain-specific *Ank3* isoforms in subsequent experiments.

**Supplementary References**

1. Zhou D, Lambert S, Malen PL, Carpenter S, Boland LM, and Bennett V. AnkyrinG is required for clustering of voltage-gated Na channels at axon initial segments and for normal action potential firing. *J Cell Biol* 1998; **143**: 1295-1304.

2. Bolger AM, Lohse M, and Usadel B. Trimmomatic: a flexible trimmer for Illumina sequence data. *Bioinformatics* 2014; **30**: 2114-2120.

3. Trapnell C, Roberts A, Goff L, Pertea G, Kim D, Kelley DR, et al. Differential gene and transcript expression analysis of RNA-seq experiments with TopHat and Cufflinks. *Nat Protoc* 2012; **7**: 562-578.

4. Marques F, Sousa JC, Coppola G, Gao F, Puga R, Brentani H, et al. Transcriptome signature of the adult mouse choroid plexus. *Fluids Barriers CNS* 2011; **8**: 10.

5. Chang DT, Honick AS, and Reynolds IJ. Mitochondrial trafficking to synapses in cultured primary cortical neurons. *J Neurosci* 2006; **26**: 7035-7045.

6. Lorenzo DN, Badea A, Davis J, Hostettler J, He J, Zhong G, et al. A PIK3C3-ankyrin-B-dynactin pathway promotes axonal growth and multiorganelle transport. *J Cell Biol* 2014; **207**: 735-752.

7. Wang X, and Schwarz TL. The mechanism of Ca2+ -dependent regulation of kinesin-mediated mitochondrial motility. *Cell* 2009; **136**: 163-174.

8. Pekkurnaz G, Trinidad JC, Wang X, Kong D, and Schwarz TL. Glucose regulates mitochondrial motility via Milton modification by O-GlcNAc transferase. *Cell* 2014; **158**: 54-68.

9. Schneider CA, Rasband WS, and Eliceiri KW. NIH Image to ImageJ: 25 years of image analysis. *Nature methods* 2012; **9**: 671-675.

10. Konermann S, Brigham MD, Trevino AE, Joung J, Abudayyeh OO, Barcena C, et al. Genome-scale transcriptional activation by an engineered CRISPR-Cas9 complex. *Nature* 2015; **517**: 583-588.

11. Kearns NA, Genga RM, Enuameh MS, Garber M, Wolfe SA, and Maehr R. Cas9 effector-mediated regulation of transcription and differentiation in human pluripotent stem cells. *Development* 2014; **141**: 219-223.

12. Sharma N, Kosan ZA, Stallworth JE, Berbari NF, and Yoder BK. Soluble levels of cytosolic tubulin regulate ciliary length control. *Mol Biol Cell* 2011; **22**: 806-816.

13. Tokesi N, Lehotzky A, Horvath I, Szabo B, Olah J, Lau P, et al. TPPP/p25 promotes tubulin acetylation by inhibiting histone deacetylase 6. *J Biol Chem* 2010; **285**: 17896-17906.
