# Supplementary material for: Disruption of the psychiatric risk gene Ankyrin 3 enhances microtubule dynamics through GSK3/CRMP2 signaling
Source: Transl Psychiatry. 2018 Jul 25;8:135. doi: 10.1038/s41398-018-0182-y (PMC6060177; doi:10.1038/s41398-018-0182-y)
Supplement: Supplementary file 3 — Supplementary Table 1 [file 41398_2018_182_MOESM3_ESM.docx]

**Supplementary Table 1.** Antibodies used for Western blot analyses.

|  | **Source** | **Species** | **Dilution** | **Catalog #** |
| --- | --- | --- | --- | --- |
| **Primary Antibodies** |  |  |  |  |
| End-binding protein 3 | Abcam | Rat | 1:2000 | ab53360 |
| α-tubulin | Cell Signaling | Mouse | 1:2000 | #3873 |
| acetyl-α-tubulin (K40) | Millipore Sigma | Mouse | 1:2000 | #T7451 |
| GSK3β | Santa Cruz | Rabbit | 1:1500 | sc9166 |
| GSK3α | Cell Signaling | Rabbit | 1:2000 | #4818S |
| GSK3β-pS9 | Cell Signaling | Rabbit | 1:1000 | #9323 |
| GSK3α-pS21 | Cell Signaling | Rabbit | 1:2000 | #9327S |
| GSK3β/α-pY216/pY279 | ABCAM | Rabbit | 1:2000 | ab4797 |
| CRMP2 | Cell Signaling | Rabbit | 1:1000 | #9393 |
| CRMP2-pT514 | Cell Signaling | Rabbit | 1:1000 | #9397 |
| GAPDH | Millipore | Rabbit | 1:2000 | ABS16 |
| **Secondary Antibodies** |  |  |  |  |
| Anti-Rabbit-HRP | Cell Signaling | Goat | 1:2000 | #7074 |
| Anti-Mouse-HRP | Cell Signaling | Goat | 1:2000 | #7076 |
| Anti-Rat-HRP | Cell Signaling | Goat | 1:2000 | #7077 |
