# Supplementary material for: Disruption of the psychiatric risk gene Ankyrin 3 enhances microtubule dynamics through GSK3/CRMP2 signaling
Source: Transl Psychiatry. 2018 Jul 25;8:135. doi: 10.1038/s41398-018-0182-y (PMC6060177; doi:10.1038/s41398-018-0182-y)
Supplement: Supplementary file 4 — Supplementary Table 2 [file 41398_2018_182_MOESM4_ESM.docx]

**Supplementary Table 2.** CRISPR/dCas9 single guide RNA information.

| **Guide RNA** | **Target Position*** | **Guide RNA Sequence (20bp)** | **PAM sequence** |
| --- | --- | --- | --- |
| sgRNA #01 | Chr10: 69706772 - 69706794 | TCTGCGCGCTGCTTGAGACA | ggg |
| sgRNA #02 | Chr10: 69706771 - 69706793 | CTCTGCGCGCTGCTTGAGAC | agg |
| sgRNA #03 | Chr10: 69706726 - 69706748 | TGCCTGATTAGAGGTACCCA | agg |
| sgRNA #04 | Chr10: 69706717 - 69706739 | GCTGCTCTATGCCTGATTAG | agg |
| sgRNA #05 | Chr10: 69706689 - 69706711 | GCTCAGGCTTCTCTCCTGCG | agg |
| sgRNA #06 | Chr10: 69706690 - 69706712 | CTCAGGCTTCTCTCCTGCGA | ggg |
| sgRNA #07 | Chr10: 69706673 - 69706695 | TCTTGGATGCCTTTCTGCTC | agg |
| sgRNA #08 | Chr10: 69706656 - 69706678 | ATCTCTGCCTCTCTGCCTCT | tgg |
| sgRNA #09 | Chr10: 69706608 - 69706630 | GATCTGGCCCCTGCTGTGGT | ggg |
| sgRNA #10 | Chr10: 69706607 - 69706629 | AGATCTGGCCCCTGCTGTGG | tgg |
| sgRNA #11 | Chr10: 69706592 - 69706614 | GCAAGGTGTAGGATGAGATC | tgg |
| sgRNA #12 | Chr10: 69706575 - 69706597 | CCAGAGGGGAAGGCTCAGCA | agg |
| sgRNA #13 | Chr10: 69706581 - 69706603 | GGGAAGGCTCAGCAAGGTGT | agg |
| sgRNA #14 | Chr10: 69706565 - 69706587 | GTGCTCTCCTCCAGAGGGGA | agg |
| sgRNA Control | No target | GAGGGTGAGCCGATCCTCCG | agg |
| *Mus GRCm38/mm10 genome assembly | |  |  |
