# Supplementary material for: Disruption of the psychiatric risk gene Ankyrin 3 enhances microtubule dynamics through GSK3/CRMP2 signaling
Source: Transl Psychiatry. 2018 Jul 25;8:135. doi: 10.1038/s41398-018-0182-y (PMC6060177; doi:10.1038/s41398-018-0182-y)
Supplement: Supplementary file 5 — Supplementary Table 3 [file 41398_2018_182_MOESM5_ESM.docx]

| **Supplementary Table 3.** qPCR primer sequences used to quantify transcript expression. | |
| --- | --- |
|  |  |
|  | **Sequence** |
| **Ank3 Primers** |  |
| Ank3 exon 1b forward | CTGCCTCCACTACAGCCTCT |
| Ank3 exon1b reverse | GGAGAGAAGCCTGAGCAGAA |
| Ank3 exon 1e forward | GAGAAGCAAGCCCTGAAATG |
| Ank3 exon 1e reverse | GGTGTTCTCTCCAGCAAAGC |
| Ank3 exon 1f forward | TCATGACCTTGTTGCAGAGC |
| Ank3 exon 1f reverse | GGCCTTACACATCGCATCTT |
| Ank3 exon 1s forward | GAGCGGTCAGAAGGATGTTC |
| Ank3 exon 1s reverse | CAAACCCGTCCCTAGTCAGA |
| **Reference Gene Primers** |  |
| beta-2-microglobulin forward | TTCTGGTGCTTGTCTCACTGA |
| beta-2-microglobulin reverse | CAGTATGTTCGGCTTCCCATTC |
