# Supplementary figures and images for: Disruption of the psychiatric risk gene Ankyrin 3 enhances microtubule dynamics through GSK3/CRMP2 signaling
Source: Transl Psychiatry. 2018 Jul 25;8:135. doi: 10.1038/s41398-018-0182-y (PMC6060177; doi:10.1038/s41398-018-0182-y)

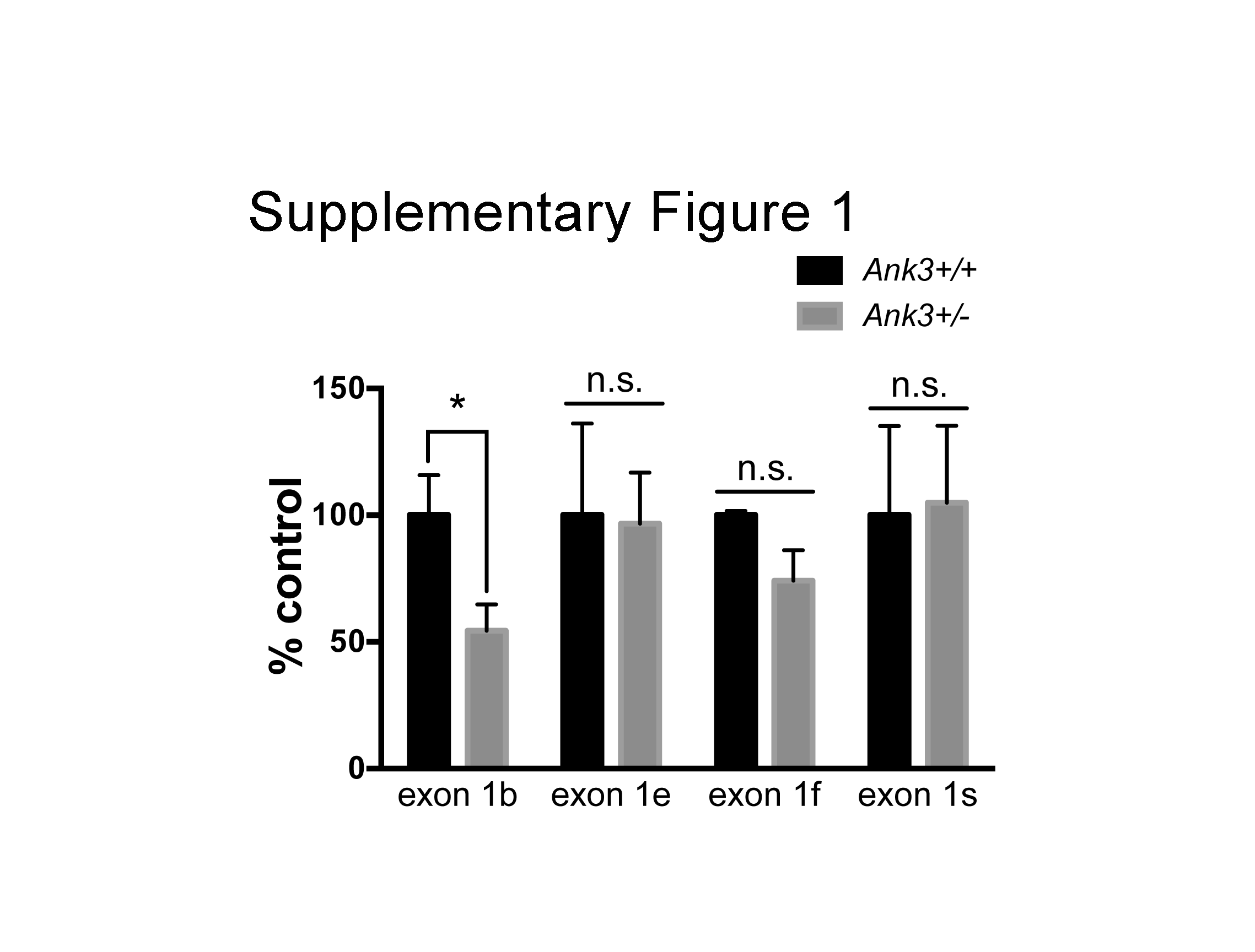

Supplement: Supplementary file 6 — Supplementary Figure 1 [file 41398_2018_182_MOESM6_ESM.tif]

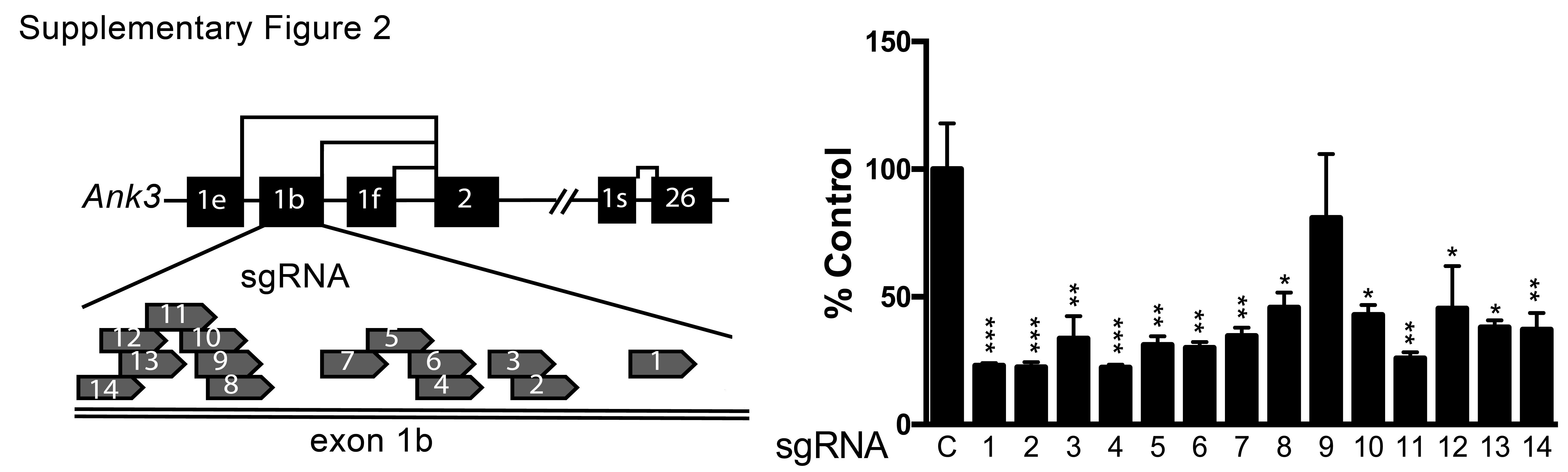

Supplement: Supplementary file 8 — Supplementary Figure 2 [file 41398_2018_182_MOESM8_ESM.tif]

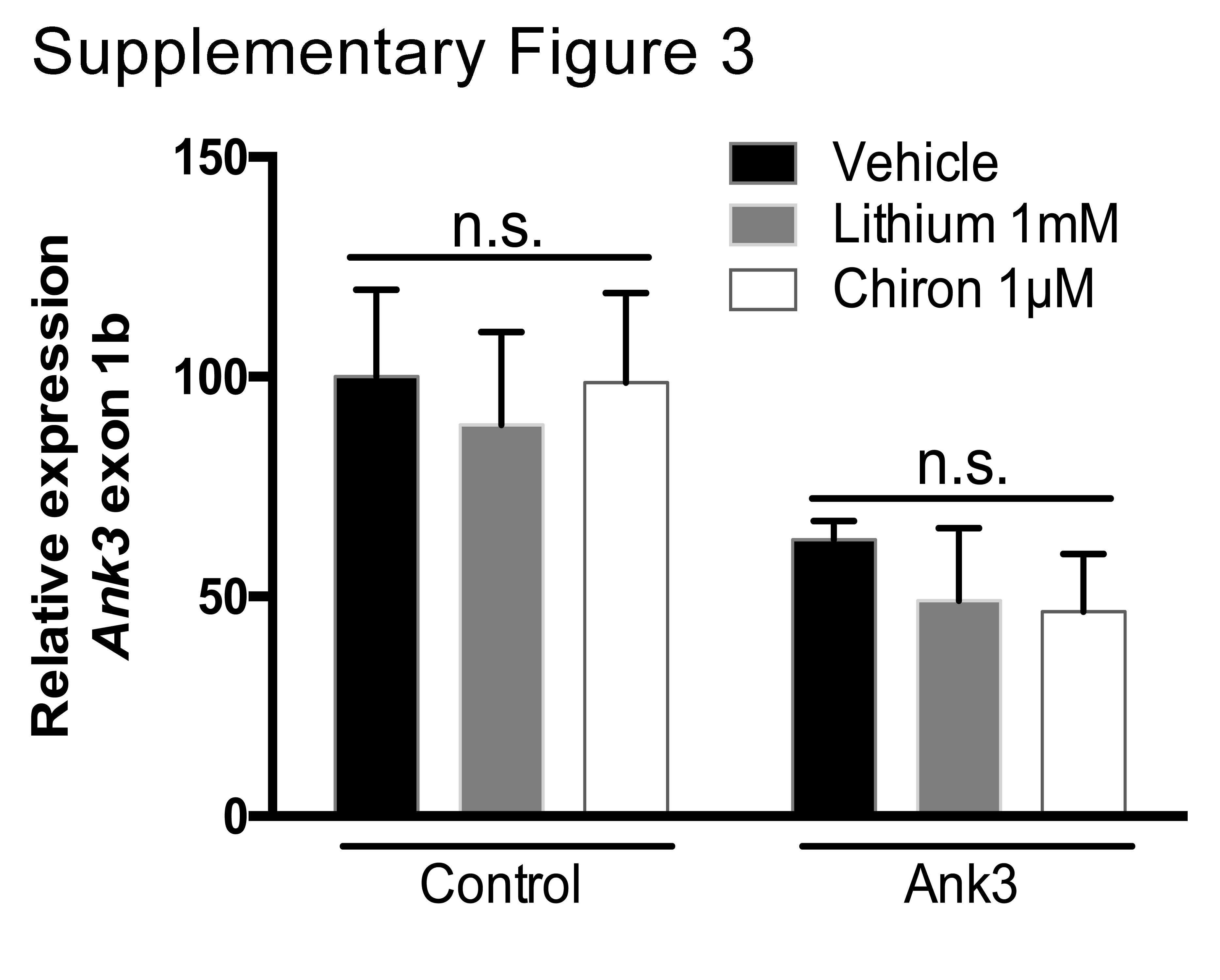

Supplement: Supplementary file 9 — Supplementary Figure 3 [file 41398_2018_182_MOESM9_ESM.tif]
