# Supplementary material for: Disruption of the psychiatric risk gene Ankyrin 3 enhances microtubule dynamics through GSK3/CRMP2 signaling
Source: Transl Psychiatry. 2018 Jul 25;8:135. doi: 10.1038/s41398-018-0182-y (PMC6060177; doi:10.1038/s41398-018-0182-y)
Supplement: Supplementary file 7 — Supplementary Table 4 [file 41398_2018_182_MOESM7_ESM.docx]

**Supplementary Table 4.** Significantly differentially expressed genes (fold change ≥1.2, *P*<10E-3) between *Ank3*+/- and *Ank3*+/+ mouse hippocampus.

| Gene | *Ank3*+/+ value | *Ank3*+/- value | *P*-value | Fold change (*Ank3*+/- vs *Ank3*+/+) |
| --- | --- | --- | --- | --- |
| Prkcd | 8.28 | 23.65 | 0 | 2.86 |
| Rps27 | 2.46 | 78.87 | 6.00E-15 | 32.10 |
| Slc17a6 | 4.13 | 8.39 | 1.44E-14 | 2.03 |
| Erdr1 | 58.51 | 30.16 | 6.93E-14 | -1.94 |
| Kl | 5.44 | 9.63 | 4.01E-13 | 1.77 |
| Eif3j2 | 11.82 | 3.55 | 3.16E-12 | -3.33 |
| 1500015O10Rik | 5.29 | 14.28 | 6.34E-09 | 2.70 |
| Wfs1 | 42.38 | 30.34 | 7.04E-09 | -1.40 |
| Plcb4 | 3.55 | 6.43 | 2.88E-08 | 1.81 |
| Plekhg1 | 2.89 | 4.90 | 8.07E-08 | 1.70 |
| Rgs16 | 3.02 | 6.36 | 1.15E-07 | 2.11 |
| Ramp3 | 3.59 | 9.01 | 1.53E-07 | 2.51 |
| Gabra4 | 23.27 | 31.53 | 2.35E-07 | 1.35 |
| Beta-s | 55.25 | 82.77 | 3.95E-07 | 1.50 |
| Pcp4l1 | 29.81 | 41.73 | 1.43E-06 | 1.40 |
| Amotl1 | 2.95 | 4.33 | 3.14E-06 | 1.47 |
| Mdga1 | 12.60 | 9.45 | 3.35E-06 | -1.33 |
| Pdp1 | 12.48 | 18.45 | 3.52E-06 | 1.48 |
| Ide | 13.54 | 9.91 | 3.97E-06 | -1.37 |
| Zmat4 | 10.85 | 14.90 | 4.83E-06 | 1.37 |
| Ap1s2 | 2.97 | 5.20 | 8.09E-06 | 1.75 |
| Cab39l | 8.93 | 12.89 | 8.44E-06 | 1.44 |
| Sowahc | 2.01 | 3.54 | 9.01E-06 | 1.76 |
| Stx1a | 58.90 | 45.57 | 1.54E-05 | -1.29 |
| Matk | 76.71 | 59.54 | 1.57E-05 | -1.29 |
| Tshz2 | 13.09 | 9.80 | 1.91E-05 | -1.34 |
| Nxph3 | 13.70 | 9.23 | 2.99E-05 | -1.48 |
| Zfr2 | 15.42 | 11.34 | 3.10E-05 | -1.36 |
| Gsg1l | 11.56 | 8.40 | 3.48E-05 | -1.38 |
| Gm3893 | 4.54 | 2.71 | 3.54E-05 | -1.68 |
| Pcp4 | 183.27 | 233.84 | 3.57E-05 | 1.28 |
| Ulk1 | 18.72 | 14.53 | 3.72E-05 | -1.29 |
| BC068157 | 10.53 | 7.77 | 4.66E-05 | -1.36 |
| Mt2 | 129.04 | 168.46 | 5.16E-05 | 1.31 |
| Trpm3 | 10.41 | 14.28 | 5.39E-05 | 1.37 |
| Arhgap33 | 34.59 | 27.53 | 6.34E-05 | -1.26 |
| Cobl | 6.24 | 4.47 | 7.60E-05 | -1.40 |
| Ccdc85c | 15.29 | 10.13 | 8.01E-05 | -1.51 |
| Myh9 | 14.83 | 11.72 | 8.35E-05 | -1.27 |
| Bcr | 26.88 | 21.56 | 8.77E-05 | -1.25 |
| Lmtk3 | 69.87 | 56.15 | 9.40E-05 | -1.24 |
| Tle2 | 7.05 | 4.45 | 9.56E-05 | -1.59 |
| Panx2 | 42.90 | 34.35 | 0.000108852 | -1.25 |
| Samd14 | 18.74 | 14.35 | 0.000113219 | -1.31 |
| Sema5a | 11.66 | 14.50 | 0.000120492 | 1.24 |
| Atn1 | 46.82 | 37.81 | 0.000128337 | -1.24 |
| Prr12 | 16.66 | 13.29 | 0.000143019 | -1.25 |
| Nat10 | 7.86 | 5.59 | 0.000156727 | -1.41 |
| Map4k2 | 16.39 | 12.14 | 0.000188572 | -1.35 |
| N4bp3 | 6.65 | 4.22 | 0.000190501 | -1.57 |
| Tbc1d8b | 2.72 | 3.96 | 0.000190929 | 1.46 |
| Plxnd1 | 7.09 | 5.39 | 0.000205085 | -1.32 |
| Ucp2 | 2.95 | 4.50 | 0.000205276 | 1.52 |
| Ttyh3 | 54.77 | 44.56 | 0.000205699 | -1.23 |
| Filip1 | 3.71 | 2.35 | 0.000223523 | -1.58 |
| Arhgap23 | 26.36 | 21.33 | 0.000224319 | -1.24 |
| Folr1 | 2.49 | 6.11 | 0.000270761 | 2.45 |
| Npas1 | 7.39 | 4.66 | 0.000287362 | -1.59 |
| Ptpru | 12.10 | 9.49 | 0.000290376 | -1.28 |
| Sstr4 | 15.97 | 10.82 | 0.00029168 | -1.48 |
| Gm5506 | 22.31 | 16.57 | 0.000292577 | -1.35 |
| Nr4a3 | 21.65 | 16.22 | 0.000337259 | -1.33 |
| Vps18 | 15.16 | 11.85 | 0.000347225 | -1.28 |
| Fbxl19 | 15.96 | 12.35 | 0.000349335 | -1.29 |
| Mpped1 | 84.04 | 68.93 | 0.000361027 | -1.22 |
| Cyp4f15 | 5.65 | 3.37 | 0.000370904 | -1.68 |
| Tpd52l1 | 13.20 | 19.05 | 0.000390124 | 1.44 |
| AI464131 | 9.28 | 6.96 | 0.000391814 | -1.33 |
| Selplg | 12.08 | 8.82 | 0.000397518 | -1.37 |
| Pitpnm3 | 9.56 | 7.52 | 0.000407994 | -1.27 |
| Gramd1a | 30.61 | 24.44 | 0.000429324 | -1.25 |
| Begain | 44.23 | 35.92 | 0.000433806 | -1.23 |
| Ptpn3 | 10.49 | 13.10 | 0.000443963 | 1.25 |
| Htr1a | 9.09 | 6.88 | 0.000449899 | -1.32 |
| D8Ertd82e | 7.03 | 5.23 | 0.00051794 | -1.34 |
| Brpf3 | 6.08 | 4.57 | 0.000520428 | -1.33 |
| Foxo6 | 10.48 | 7.52 | 0.000527131 | -1.39 |
| Prps2 | 7.12 | 9.53 | 0.000536416 | 1.34 |
| Ntng1 | 7.38 | 10.34 | 0.00054272 | 1.40 |
| Col4a2 | 8.86 | 6.96 | 0.000550112 | -1.27 |
| Tyro3 | 30.90 | 25.23 | 0.000551105 | -1.22 |
| Ptpn4 | 6.34 | 8.36 | 0.000574371 | 1.32 |
| Megf8 | 22.84 | 18.88 | 0.000611696 | -1.21 |
| Avpi1 | 9.57 | 15.16 | 0.000638647 | 1.58 |
| Rgs11 | 6.97 | 4.43 | 0.000683237 | -1.57 |
| Plxna1 | 23.24 | 19.24 | 0.000686438 | -1.21 |
| Mll2 | 8.21 | 6.77 | 0.000692648 | -1.21 |
| Ttbk1 | 18.50 | 15.19 | 0.000696893 | -1.22 |
| Plch2 | 25.00 | 20.57 | 0.000702597 | -1.21 |
| Cgnl1 | 2.52 | 3.52 | 0.000738458 | 1.40 |
| Nr1d2 | 15.27 | 18.85 | 0.000764468 | 1.23 |
| Gm12191 | 246.37 | 303.87 | 0.000783589 | 1.23 |
| Sipa1l3 | 26.76 | 22.21 | 0.000816567 | -1.21 |
| BC037034 | 24.53 | 19.41 | 0.000855367 | -1.26 |
| Kcnip4 | 20.96 | 26.83 | 0.000877353 | 1.28 |
| Bahd1 | 13.65 | 10.85 | 0.000888779 | -1.26 |
| Gpr124 | 3.54 | 2.46 | 0.000904067 | -1.44 |
| Dact3 | 47.79 | 39.44 | 0.000922353 | -1.21 |
| Shisa7 | 30.97 | 25.73 | 0.000952881 | -1.20 |
| Hbb-b2 | 8.24 | 16.11 | 0.000953578 | 1.95 |
| Ncor2 | 26.20 | 21.63 | 0.000961231 | -1.21 |
| Slc36a1 | 13.53 | 10.91 | 0.00101608 | -1.24 |
| Cecr6 | 12.67 | 10.12 | 0.00101723 | -1.25 |
| Shank1 | 69.39 | 57.27 | 0.00101991 | -1.21 |
| Igfbp2 | 18.18 | 24.37 | 0.00102307 | 1.34 |
| Zfp71-rs1 | 2.13 | 3.31 | 0.00103424 | 1.56 |
| Stx8 | 2.64 | 3.92 | 0.00104583 | 1.49 |
| Ahdc1 | 10.49 | 8.45 | 0.00108993 | -1.24 |
| Rn45s | 172.93 | 139.39 | 0.00110034 | -1.24 |
| Cul9 | 11.69 | 9.56 | 0.00110769 | -1.22 |
| Rras2 | 5.05 | 7.44 | 0.0011085 | 1.47 |
| Meis2 | 12.95 | 10.06 | 0.0011247 | -1.29 |
| Oplah | 8.34 | 6.29 | 0.00114626 | -1.33 |
| Edil3 | 24.44 | 29.38 | 0.0011798 | 1.20 |
| Cacng5 | 2.35 | 3.93 | 0.00118831 | 1.68 |
| Wnk2 | 18.09 | 14.97 | 0.00128617 | -1.21 |
| Zmiz2 | 61.92 | 50.83 | 0.00134548 | -1.22 |
| Flnb | 7.64 | 6.18 | 0.00135148 | -1.24 |
| Abcb8 | 13.48 | 10.43 | 0.00136421 | -1.29 |
| Fam84b | 2.88 | 4.02 | 0.00141246 | 1.40 |
| Abcc8 | 7.69 | 5.95 | 0.00142273 | -1.29 |
| Maoa | 6.52 | 8.50 | 0.00147613 | 1.30 |
| Aldh6a1 | 17.53 | 21.75 | 0.00149156 | 1.24 |
| Srrd | 14.03 | 8.58 | 0.00155992 | -1.63 |
| Mllt1 | 24.01 | 19.70 | 0.00158019 | -1.22 |
| Axin2 | 4.13 | 5.62 | 0.00177193 | 1.36 |
| Fmnl1 | 33.16 | 27.52 | 0.00178177 | -1.21 |
| Lhx2 | 29.00 | 23.48 | 0.00179915 | -1.23 |
| Enthd2 | 5.59 | 3.81 | 0.00181962 | -1.47 |
| Tmed7 | 26.50 | 31.92 | 0.00182075 | 1.20 |
| Dvl3 | 12.00 | 9.25 | 0.00184001 | -1.30 |
| Mmgt1 | 8.33 | 10.59 | 0.00185902 | 1.27 |
| Nat14 | 42.33 | 34.31 | 0.00186634 | -1.23 |
| Cacna1a | 19.81 | 16.36 | 0.00191367 | -1.21 |
| Rfc3 | 2.23 | 4.46 | 0.00191478 | 2.00 |
| Erlec1 | 9.77 | 12.41 | 0.00194774 | 1.27 |
| Asphd2 | 56.73 | 46.69 | 0.00195144 | -1.22 |
| Dvl2 | 6.68 | 4.76 | 0.00196175 | -1.40 |
| Zfp668 | 4.36 | 2.97 | 0.00204139 | -1.47 |
| Zfp90 | 4.13 | 2.61 | 0.00211142 | -1.58 |
| Scrib | 8.00 | 6.33 | 0.00212052 | -1.26 |
| Slc12a2 | 10.36 | 12.58 | 0.00212879 | 1.21 |
| Tmem198 | 26.42 | 21.28 | 0.00216262 | -1.24 |
| Bsn | 37.53 | 31.27 | 0.00222674 | -1.20 |
| Zfp943 | 3.96 | 5.76 | 0.00224377 | 1.45 |
| Nr4a2 | 21.60 | 17.20 | 0.00235163 | -1.26 |
| Dsp | 3.63 | 4.57 | 0.00238398 | 1.26 |
| Trim11 | 9.61 | 7.00 | 0.00238814 | -1.37 |
| Asnsd1 | 5.41 | 7.65 | 0.00239157 | 1.41 |
| Chrac1 | 6.14 | 10.46 | 0.00244816 | 1.70 |
| 2010012O05Rik | 6.88 | 9.51 | 0.00245851 | 1.38 |
| Cav2 | 10.30 | 13.37 | 0.0024853 | 1.30 |
| Fcho1 | 19.44 | 15.16 | 0.00249066 | -1.28 |
| Fam107b | 3.47 | 5.01 | 0.00250129 | 1.44 |
| Otud7a | 14.26 | 11.42 | 0.0025165 | -1.25 |
| Ttc9b | 63.33 | 50.82 | 0.00255056 | -1.25 |
| Nuak1 | 15.35 | 12.65 | 0.00257166 | -1.21 |
| Lrrc55 | 2.76 | 4.21 | 0.00257501 | 1.52 |
| Clmp | 7.11 | 5.17 | 0.00259943 | -1.37 |
| Slit3 | 15.59 | 12.86 | 0.00262506 | -1.21 |
| Il34 | 26.83 | 21.29 | 0.00264375 | -1.26 |
| Sept2 | 13.16 | 16.58 | 0.00270163 | 1.26 |
| Phf1 | 17.35 | 13.72 | 0.00274904 | -1.26 |
| Rtfdc1 | 36.42 | 30.01 | 0.00278548 | -1.21 |
| Hcn3 | 3.93 | 2.63 | 0.00284249 | -1.50 |
| Kcnj13 | 2.19 | 4.88 | 0.00284297 | 2.23 |
| Vps72 | 18.25 | 13.67 | 0.00289064 | -1.34 |
| Plod3 | 10.44 | 7.89 | 0.00299979 | -1.32 |
| Kcnc4 | 24.36 | 20.07 | 0.00301791 | -1.21 |
| Ankrd27 | 9.58 | 7.36 | 0.00312409 | -1.30 |
| Tmem189 | 22.60 | 17.75 | 0.00313121 | -1.27 |
| Prrt3 | 15.52 | 12.53 | 0.00314022 | -1.24 |
| Gas2l1 | 21.17 | 17.00 | 0.00315149 | -1.25 |
| Etf1 | 19.90 | 23.92 | 0.00315928 | 1.20 |
| Fa2h | 13.64 | 10.62 | 0.00323896 | -1.28 |
| Nt5c3l | 13.56 | 18.55 | 0.00324144 | 1.37 |
| Klhl34 | 27.55 | 22.27 | 0.00328047 | -1.24 |
| Cacng3 | 39.40 | 32.07 | 0.00332411 | -1.23 |
| Dok3 | 4.35 | 2.47 | 0.00343933 | -1.76 |
| Ptprt | 9.23 | 7.55 | 0.00359965 | -1.22 |
| Lypla1 | 8.60 | 11.28 | 0.00359981 | 1.31 |
| Cdk18 | 8.96 | 6.84 | 0.00366674 | -1.31 |
| Taf6l | 3.53 | 2.08 | 0.0036979 | -1.70 |
| Bcar1 | 20.11 | 15.94 | 0.00376529 | -1.26 |
| Stxbp2 | 7.47 | 5.48 | 0.00377942 | -1.36 |
| Slc6a7 | 22.69 | 18.81 | 0.00379198 | -1.21 |
| Sh3d19 | 3.08 | 4.05 | 0.00387293 | 1.31 |
| E2f4 | 15.60 | 12.08 | 0.00389815 | -1.29 |
| Gse1 | 16.71 | 13.71 | 0.00398018 | -1.22 |
| Phrf1 | 9.63 | 7.83 | 0.00403921 | -1.23 |
| Pde4a | 28.06 | 23.10 | 0.00413555 | -1.21 |
| Trmu | 6.89 | 4.44 | 0.00415623 | -1.55 |
| Gpr123 | 14.97 | 12.41 | 0.00420187 | -1.21 |
| Arsg | 5.98 | 8.03 | 0.00421158 | 1.34 |
| Osbpl7 | 5.11 | 3.70 | 0.00422112 | -1.38 |
| Pcolce | 2.96 | 4.96 | 0.00426033 | 1.68 |
| Fbxo42 | 6.96 | 5.58 | 0.00427933 | -1.25 |
| Map2k2 | 25.98 | 21.35 | 0.00428508 | -1.22 |
| Xrcc1 | 12.09 | 9.20 | 0.00430008 | -1.31 |
| Vimp | 19.81 | 25.69 | 0.00431409 | 1.30 |
| AI504432 | 4.44 | 5.68 | 0.00433923 | 1.28 |
| Tril | 11.61 | 9.57 | 0.00434556 | -1.21 |
| Zfp358 | 12.12 | 9.14 | 0.00436105 | -1.33 |
| E030003E18Rik | 5.42 | 8.74 | 0.00439864 | 1.61 |
| Cox20 | 21.91 | 33.22 | 0.00447466 | 1.52 |
| Rims4 | 16.42 | 11.07 | 0.00460363 | -1.48 |
| Rhbdl1 | 30.96 | 25.00 | 0.00460742 | -1.24 |
| Pcif1 | 16.06 | 12.93 | 0.00461411 | -1.24 |
| Zfp825 | 3.26 | 5.46 | 0.00471187 | 1.68 |
| Limk1 | 13.92 | 11.27 | 0.0048623 | -1.24 |
| Ajap1 | 15.22 | 12.33 | 0.00490859 | -1.23 |
| Bag3 | 4.56 | 3.07 | 0.00495702 | -1.48 |
| Nr1h2 | 19.26 | 15.32 | 0.0050242 | -1.26 |
| Arx | 5.22 | 3.65 | 0.00510946 | -1.43 |
| Gsta4 | 39.22 | 48.74 | 0.00516525 | 1.24 |
| Rpia | 2.64 | 4.34 | 0.00517519 | 1.65 |
| Gmds | 7.61 | 5.07 | 0.00524912 | -1.50 |
| Setd1b | 6.51 | 5.36 | 0.00525235 | -1.21 |
| Churc1 | 23.35 | 32.26 | 0.00529914 | 1.38 |
| Ephb6 | 21.64 | 18.02 | 0.00548914 | -1.20 |
| Gpr176 | 4.11 | 2.96 | 0.0055724 | -1.39 |
| Tgfb3 | 3.35 | 2.26 | 0.00558701 | -1.48 |
| Rfx3 | 6.68 | 8.24 | 0.00560877 | 1.23 |
| 1110004E09Rik | 16.55 | 21.78 | 0.00566411 | 1.32 |
| Spred3 | 13.52 | 11.12 | 0.00576774 | -1.22 |
| Rexo4 | 14.55 | 11.49 | 0.0058474 | -1.27 |
| Ppid | 32.74 | 39.39 | 0.00590395 | 1.20 |
| Wsb1 | 21.28 | 16.58 | 0.00600634 | -1.28 |
| Nxt2 | 11.07 | 13.97 | 0.00600993 | 1.26 |
| Cckbr | 6.30 | 4.51 | 0.00601234 | -1.40 |
| C330018D20Rik | 3.21 | 4.75 | 0.00613148 | 1.48 |
| Lmo2 | 7.51 | 11.22 | 0.00640978 | 1.49 |
| Agt | 10.77 | 14.00 | 0.00642589 | 1.30 |
| Npas2 | 7.13 | 5.60 | 0.00646178 | -1.27 |
| Phf14 | 7.88 | 9.94 | 0.00680172 | 1.26 |
| Mcm3ap | 9.62 | 7.97 | 0.0068228 | -1.21 |
| Pdrg1 | 16.09 | 11.92 | 0.00703838 | -1.35 |
| Grb14 | 13.53 | 10.49 | 0.00706948 | -1.29 |
| Olfm3 | 4.89 | 6.58 | 0.00710646 | 1.34 |
| Trmt61b | 3.07 | 6.30 | 0.00713782 | 2.05 |
| Rpl34,Rpl34-ps1 | 25.88 | 13.36 | 0.00714877 | -1.94 |
| E130012A19Rik | 15.93 | 12.79 | 0.00718956 | -1.25 |
| Cpsf1 | 13.64 | 11.21 | 0.00732394 | -1.22 |
| Gpr116 | 4.10 | 5.02 | 0.00739527 | 1.23 |
| Vat1l | 7.88 | 9.82 | 0.00740899 | 1.25 |
| Wnt7a | 5.11 | 3.73 | 0.0075324 | -1.37 |
| Vwa5b2 | 6.22 | 4.89 | 0.00762056 | -1.27 |
| Tmem63c | 12.18 | 9.89 | 0.00773783 | -1.23 |
| Plcg1 | 14.51 | 11.85 | 0.00775173 | -1.22 |
| Tarbp2 | 8.92 | 6.45 | 0.00786247 | -1.38 |
| Golt1b | 8.59 | 10.85 | 0.00790952 | 1.26 |
| Aldh1a1 | 17.16 | 21.09 | 0.00802402 | 1.23 |
| Crem | 4.16 | 6.50 | 0.00814194 | 1.56 |
| Ctdspl2 | 4.48 | 5.74 | 0.00816269 | 1.28 |
| Srrt | 31.15 | 25.73 | 0.00819871 | -1.21 |
| Cox19 | 30.20 | 22.76 | 0.00820211 | -1.33 |
| Ube2d1 | 23.45 | 28.93 | 0.00821705 | 1.23 |
| Chst12 | 12.32 | 9.38 | 0.00823839 | -1.31 |
| Tceal8 | 14.41 | 17.79 | 0.00836168 | 1.23 |
| Pole3 | 9.64 | 15.36 | 0.00838321 | 1.59 |
| Dpysl5 | 6.34 | 5.06 | 0.00841721 | -1.25 |
| Lamp2 | 19.79 | 24.01 | 0.00843815 | 1.21 |
| Smurf1 | 8.92 | 7.04 | 0.00847586 | -1.27 |
| Pno1 | 10.87 | 14.27 | 0.00849418 | 1.31 |
| Hpcal1 | 32.67 | 26.92 | 0.00850969 | -1.21 |
| Myo9b | 5.90 | 4.67 | 0.00865463 | -1.26 |
| Szt2 | 4.85 | 4.02 | 0.00872169 | -1.21 |
| Lrrc45 | 11.99 | 9.48 | 0.00874545 | -1.26 |
| Slc30a1 | 6.46 | 7.92 | 0.00878263 | 1.23 |
| Naa30 | 7.02 | 8.63 | 0.00878795 | 1.23 |
| Fam136a | 10.27 | 13.50 | 0.00897518 | 1.31 |
| Vac14 | 8.90 | 6.98 | 0.00901558 | -1.27 |
| Hdac6 | 12.11 | 9.64 | 0.00917237 | -1.26 |
| Tmem201 | 21.17 | 17.60 | 0.00923124 | -1.20 |
| Unc5b | 6.19 | 5.01 | 0.00924916 | -1.24 |
| Cc2d2a | 4.47 | 3.48 | 0.0092672 | -1.28 |
| Gm13363 | 22.95 | 27.85 | 0.00948939 | 1.21 |
| Dot1l | 8.92 | 7.41 | 0.0095947 | -1.21 |
| Cabp1 | 46.18 | 38.27 | 0.00966034 | -1.21 |
| Ccdc64 | 12.62 | 9.75 | 0.00992582 | -1.29 |
| Qpct | 4.50 | 6.42 | 0.0099517 | 1.43 |
| Zfp935 | 2.08 | 3.35 | 0.00997528 | 1.61 |
